# Lower Urinary Tract Symptoms, Depression, Anxiety and Systemic Inflammatory Factors in Men: A Population-Based Cohort Study

**DOI:** 10.1371/journal.pone.0137903

**Published:** 2015-10-07

**Authors:** Sean Martin, Andrew Vincent, Anne W. Taylor, Evan Atlantis, Alicia Jenkins, Andrzej Januszewski, Peter O’Loughlin, Gary Wittert

**Affiliations:** 1 Freemasons Foundation Centre for Men’s Health, University of Adelaide, Adelaide, South Australia, Australia; 2 School of Medicine, University of Adelaide, Adelaide, South Australia, Australia; 3 Population Research and Outcome Studies, University of Adelaide, Adelaide, South Australia, Australia; 4 School of Nursing and Midwifery, University of Western Sydney, Sydney, New South Wales, Australia; 5 NHMRC Clinical Trials Centre, University of Sydney, Camperdown, New South Wales, Australia; 6 Chemical Pathology, SA Pathology, Adelaide, South Australia, Australia; University of Stellenbosch, SOUTH AFRICA

## Abstract

**Background:**

The relationship between lower urinary tract symptoms (LUTS) and common mental health disorders such as depression and anxiety in men remains unclear. Inflammation has recently been identified as an independent risk factor for LUTS and depression. This study aimed to assess the association between depression, anxiety and LUTS, and the moderating influence of systemic inflammation, in the presence of other biopsychosocial confounders.

**Methods:**

Participants were randomly-selected from urban, community-dwelling males aged 35–80 years at recruitment (n = 1195; sample response rate:67.8%). Of these, 730 men who attended baseline (2002–5) and follow-up clinic visits (2007–10), with complete outcome measures, and without prostate or bladder cancer and/or surgery, neurodegenerative conditions, or antipsychotic medications use, were selected for the present study. Unadjusted and multi-adjusted regression models of incident storage and voiding LUTS and incident depression and anxiety were combined with serum inflammatory markers (high-sensitive C-reactive protein (hsCRP), tumor necrosis factor-alpha (TNF-α), interleukin–6 (IL–6), myeloperoxidase (MPO), soluble e-selectin (e-Sel)) and socio-demographic, lifestyle, and health-related factors. Hierarchical multiple regression was used to assessed the moderating effect of inflammatory markers.

**Results:**

The incidence of storage, voiding LUTS, depression and anxiety was 16.3% (n = 108), 12.1% (n = 88), 14.5% (n = 108), and 12.2% (n = 107). Regression models demonstrated that men with depression and anxiety at baseline were more likely to have incident storage, but not voiding LUTS (OR: 1.26, 99%CI: 1.01–4.02; and OR:1.74; 99%CI:1.05–2.21, respectively). Men with anxiety and storage LUTS at baseline were more likely to have incident depression (OR: 2.77, 99%CI: 1.65–7.89; and OR:1.45; 99%CI:1.05–2.36, respectively), while men with depression and voiding LUTS were more likely to have anxiety at follow-up (OR: 5.06, 99%CI: 2.81–9.11; and OR:2.40; 99%CI:1.16–4.98, respectively). CRP, TNF-α, and e-Sel were found to have significant moderating effects on the development of storage LUTS (1.06, 0.91–1.96, R^2^ change: 12.7%), depression (1.17, 1.01–1.54, R^2^ change: 9.8%), and anxiety (1.35, 1.03–1.76, R^2^ change: 10.6%), respectively.

**Conclusions:**

There is a bidirectional relationship between storage, but not voiding, LUTS and both depression and anxiety. We observed variable moderation effects for selected inflammatory markers on the development of depression, anxiety and storage LUTS.

## Introduction

Lower urinary tract symptoms (LUTS) can be broadly classified as storage (increased frequency and/or urgency of micturition, and nocturia) and voiding (incomplete emptying, intermittent and/or weak stream, and straining during micturition) symptoms. The prevalence of LUTS in community-based men ranges from 13–47% of adult males [1]. Storage symptoms are more common than voiding symptoms (13–42% vs. 6–22% of adult males, respectively [1]). LUTS has been demonstrated to have an equivalent or greater impact on health-related quality of life (HR-QoL) as other major chronic diseases, such as heart disease, diabetes, and cancer [2]. Storage symptoms (especially nocturia) in particular seem to adversely impact HR-QoL, while voiding symptoms are associated with elevated distress [1].

Common mental health disorders, such as depression and anxiety, have similarly adverse impacts on HR-QoL [3]. Recent global estimates indicate that depressive and anxiety disorders were the second and sixth leading cause of years lived with disability (YLDs) [3].

While LUTS has traditionally been thought to be solely related to deteriorating bladder function or prostate abnormalities, recent studies have demonstrated associations between LUTS and obesity, type 2 diabetes, sleep disorders, arthritis, employment and marital status, testosterone, smoking and low physical activity, and medication usage (see [4] for review). Accordingly, LUTS may be indicative of systemic disease, occurring beyond, but impacting on the lower urinary tract.

Cross-sectional [5] and longitudinal studies [6] have demonstrated an independent association between the development depression and anxiety and LUTS in men. However, no study to date has distinguished between LUTS symptom type, an important distinction given the differing risk factors for storage and voiding LUTS [7]. Systemic inflammation in particular has been identified as an independent risk factor for both LUTS [8] and depression /anxiety [9] in ageing men. C-reactive protein (CRP), interleukin–6 (IL–6), and tumor necrosis factor alpha (TNF-α) have previously been shown to associate with LUTS, depression and anxiety [8] [9], while myeloperoxidase (MPO) has also been linked to the development of depression [9]. The extent to which this is independent of confounders common to both LUTS and depression /anxiety (e.g. obesity, diabetes, arthritis, smoking and alcohol consumption, sedentary behaviour, sleep disorders and medication usage) remains to be determined.

We examine the relationships between LUTS, depression, anxiety, and serum inflammatory markers in a community-dwelling cohort of middle-aged to elderly men.

## Methods

### Study design and sampling

Data were obtained from the Florey Adelaide Male Ageing Study (FAMAS), a population-based prospective cohort study of randomly-selected men from the northern and western suburbs of Adelaide, Australia [10]. A total of 1,620 men aged 35–80 years at recruitment completed a telephone interview (sample response rate = 67.8%) and 1,195 attended a clinic visit (T1; clinic response rate = 45.1%) between 2002 and 2005. Comparisons to the 2001 Australian Census data showed that FAMAS participants matched the population for most key demographics, although younger groups and never-married men were under-represented and older participants were over-represented [10]. Follow-up clinic visits using identical protocols were conducted between 2007 and 2010 (T2; n = 899), as near as practical to five years post the subjects initial visit (mean follow-up period 5.0 ± 0.2 years). Comparison to the 2006 Australian Census data showed that FAMAS participants were more likely to be older, married and have a higher level of post-school education [10].

All protocols were approved by the Royal Adelaide Hospital Research Ethics Committee, with written, informed consent obtained from all participants. This work was funded through the Australian National Health and Medical Research Council (Project Grant #627227).

### Predictor and outcome variables

#### Lower urinary tract symptoms (LUTS)

The seven-item American Urology Association–Symptom Index (AUA-SI) was used to evaluate the presence of LUTS [11]. Subjects were classified as having storage symptoms if the sum of their score on AUA-SI items 2, 4 and 7 was ≥ 4 (and their score on item 4 (urgency) was ≥ 1) and having voiding symptoms if the sum of their score on AUA-SI items 1, 3, 5 and 6 was ≥ 5.

#### Combined depression and anxiety

Depressive symptoms were assessed using the Beck Depression Inventory (BDI) [12] and anxiety symptoms were assessed using the Generalised Anxiety Disorder – 7 (GAD–7) [13]. Classification of symptomatic depression was by BDI scores of ≥ 12, and symptomatic anxiety was by GAD–7 ≥ 10. Previously diagnosed depression and anxiety were determined by self-report. Current prescriptions (within 6 months of clinic visit) for antidepressants and anxiolytics were determined by record linkage with the national Pharmaceutical Benefits Schemes (PBS) database. Classification of ‘Combined Depression’ or ‘Combined Anxiety’ required the presence of one or more of the following conditions: (1) symptomatic depression or anxiety (current symptoms), or (2) current prescription for antidepressant or anxiolytics medication, or (3) previously diagnosed depression or anxiety.

### Moderating variables

#### Inflammatory markers

C-reactive protein (CRP) was quantitated using a Cobas Integra automated clinical chemistry analyzer (Roche Diagnostics, Basel, Switzerland). Tumour necrosis factor-alpha (TNF-a), and interleukin 6 (IL–6) was quantitated with an enzyme-linked immunosorbent assay (ELISA) (R&D, Minneapolis, MN). The inter-assay CVs were 2.1% for CRP, 10.6% for TNF-α, and 7.8% for IL–6. All inflammatory markers were analysed in the same laboratory with established QC protocols [10].

### Covariate variables

#### Demographic factors, health status & medication usage

Information on age, education, marital, occupational, smoking and disease status was obtained by self-report questionnaire [10]. Medication use was determined by self-report and data linkage with the national medication registry. Obstructive sleep apnea (OSA) was defined as men with an apnea-hypopnea index (AHI) of 10 or greater based on overnight polysomnography testing.

#### Serum assays

Serum samples were drawn between 8 and 11am after a 12-hour overnight fast. Following venepuncture, samples were immediately placed in an ice slurry for no more than 3 hours before being immediately processed for long-term storage at -80C. Serum total testosterone (TT) was measured by a validated stable-isotope dilution LC–MS/MS (inter-assay CV: 9.3% at 0.43 nmol/L; 8.6% at 1.66 nmol/L, 4.0% at 8.17 nmol/L) and estradiol (E_2_) (inter-assay CV: 14% at 23 pmol/L; 4.0% at 83 pmol/L, 6.0% at 408 pmol/L). HDL cholesterol and triglycerides (TG) were measured enzymatically using a Hitachi 911, with LDL cholesterol calculated using the Friedwald equation (LDL = Total cholesterol (TC)—HDL—TG/5.0) (Boehringer, Germany; inter-assay CV: triglyceride 3%, total cholesterol 2.3%, HDL 6.7% and LDL 3.7%). Plasma glucose was determined using an automated chemistry analyser system (Olympus AU5400, Japan; inter-assay CV: 2.5% at 3.5 mmol/L and 3.0% at 19.6 mmol/L). Glycated haemoglobin (HbA1c) was measured by high-pressure liquid chromatography (HPLC) using a spherical cation exchange gel (CV 2% at 6% of total haemoglobin).

#### Body composition

Anthropometric measures, blood pressure, grip strength and body composition (by dual energy x-ray absorptiometry (DEXA)) were obtained as previously published [10].

#### Cognitive testing

Given the close association between cognitive function and anxiety / depression, measures were included that assessed episodic memory function (Fuld Object Memory Evalutation) and visual attention and task switching (Trail Making Tests A & B) [10].

#### Statistical analyses

**Fig 1:**
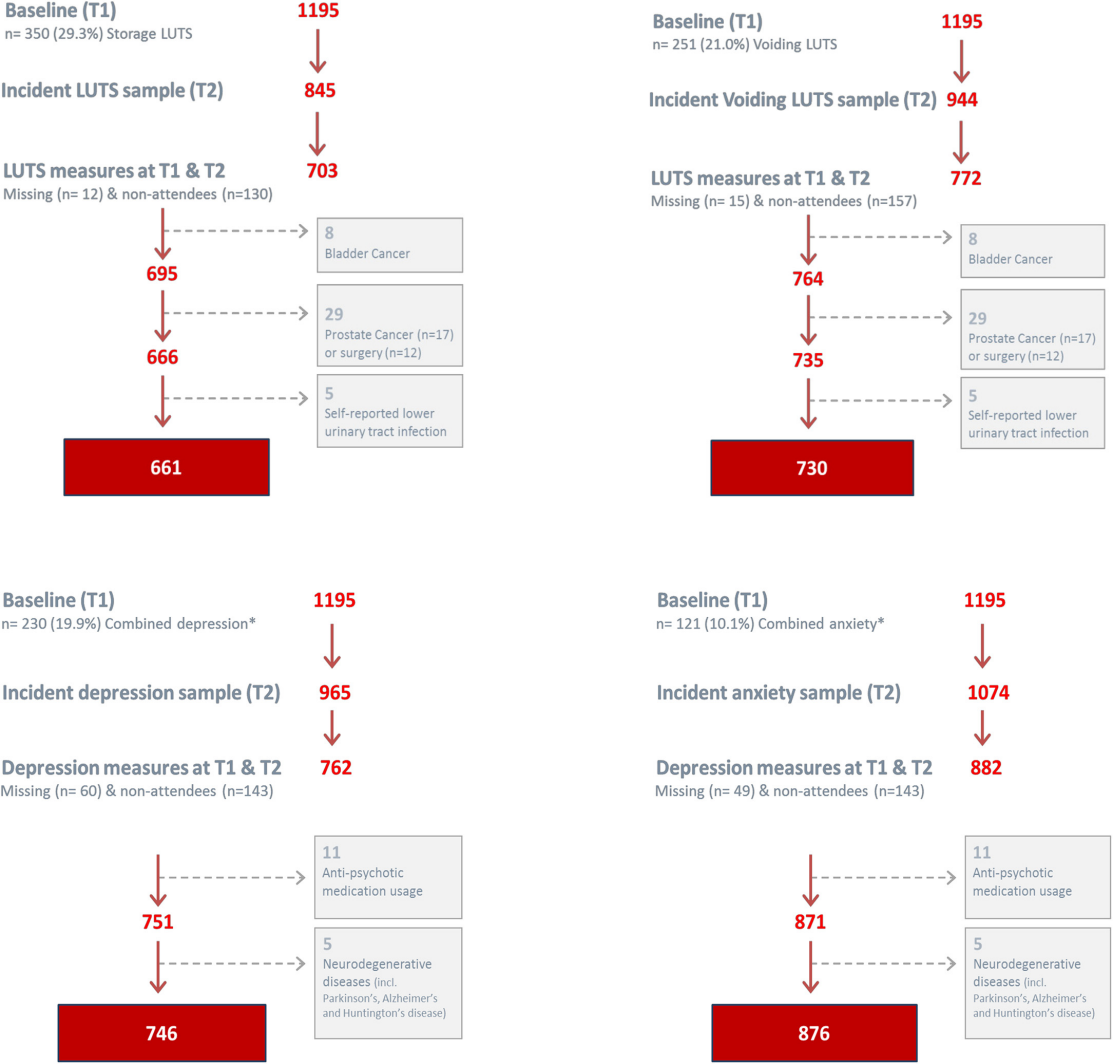
Consort diagrams for the final analytical samples for incident storage, voiding LUTS, depression and anxiety in a community cohort of Australian men.

For the present study, only men who had completed the storage and voiding LUTS questions from the AUA-SI, and all depression / anxiety measures, at T1 & T2 (n = 703, 772 & n = 698, respectively) were included. Based on an initial disease-free incidence of 25% and an exposure prevalence of approximately 15%, this sample size is sufficient to detect an effect size of between 2.0–3.5 with 99% power. Men with a history of bladder (n = 8) or prostate cancer (n = 17) or prostate surgery (n = 12), and a current self-reported urinary tract infection (n = 5) were excluded from the LUTS analysis, while men who were currently (≤ 6 months of clinic visit) using anti-psychotic medications (n = 13) or reported having a neurodegenerative condition (n = 5) were excluded from the depression and anxiety analysis (Fig 1).

Univariable logistic regression assessed the association between covariates and incident storage, voiding LUTS, depression and anxiety. For the multivariable logistic regression models, independents were first selected on the basis of demonstrated or suspected associations with the outcome. Those predictors with a unadjusted association with the outcome variable of *p* ≤0.25 were included in the final regression model. Due to multiple regression, the alpha level was placed at 0.01.

Hierarchical multiple regression was used to assess the moderating influence of all those inflammatory markers with an unadjusted regression estimate on the outcome of p<0.25. All predictor variables in these models were centred, with interaction terms included for those with an *a priori* association (e.g. storage and voiding LUTS, HDL and LDL cholesterol, age and BMI).

Data were analysed using PASW Statistics 20.0 (SPSS Inc. Chicago, USA), and forest plots maps modelled by AMOS (SPSS Inc. Chicago, USA).

## Results

Table 1 details the selected demographic and clinical variables of the analytic sample at baseline and follow-up. At follow-up, men were found to have lower handgrip strength, less full-time employment and higher rates of retirement, have a lower gross household income, a lower serum testosterone, higher proportion of sleep apnea, erectile dysfunction, lower solitary and dyadic sexual desire, and a higher proportion of men with diabetes (Table 1)

**Table 1:** Baseline (2002–5) and follow-up (2007–10) descriptive characteristics of analytic sample.

	Baseline		Follow-up		
	% / x	N / SD	% / x	N / SD	*p*
Age (years)	55	11	60	11	**0.001**
BMI (kg/m^2^)	28.5	4.3	28.8	4.5	0.432
Abdominal fat mass ^*Δ*^ (%;DEXA)	33.6	7.7	34.9	7.6	0.254
Hand grip strength *(Dominant; Nm)	49.7	9.5	42.5	9.7	**0.031**
Systolic BP (mmHg)	137.1	17.71	136.3	16.5	0.212
Diastolic BP (mmHg)	85.4	8.82	84.2	9.73	0.551
Marital status					0.497
Married / Partner	83.9%	733	82.7%	691	
Separated / Divorced	9.2%	80	8.9%	74	
Widowed	2.5%	22	3.2%	27	
Never married	4.5%	39	4.3%	36	
Work status					**0.021**
Full time	56.1%	491	46.7%	388	
Part time / Casual	8.9%	78	9.4%	78	
Unemployed	2.1%	18	2.2%	18	
Retired	25.9%	227	35.9%	298	
Educational status					0.373
Bachelor or higher	13.9%	122	18.6%	118	
Trade/Apprenticeship	33.7%	295	36.7%	296	
Certificate/Diploma	25.6%	224	33.1%	210	
Other	25.9%	227	15.0%	105	
Household Income					**0.044**
Low	30.4%	262	32.6%	273	
Middle	34.8%	308	32.9%	275	
High	33.9%	292	28.1%	235	
Leisure time physical activity					0.471
150 mins or more	34.8%	308	34.3%	280	
1–149 mins	32.6%	287	36.3%	294	
None	24.6%	214	29.4%	200	
Smoking status (current)					0.080
Yes	17.2%	151	14.1%	118	
No	79.6%	697	84.3%	707	
Triglycerides (mmol/L)	1.68	1.19	1.74	1.59	0.232
LDL chol. (mmol/L)	3.5	.9	3.1	1.0	0.411
HDL chol. (mmol/L)	1.2	.3	1.3	.3	0.738
Total T (nmol/L)	17.3	5.8	16.1	5.7	**0.050**
DHT	1.72	.74	1.65	.69	0.111
SHBG (nmol/L)	34.5	14.8	38.5	16.2	0.151
E_2_ (pmol/L)	94.7	36.3	91.8	33.8	0.422
T3 (pmol/L)	4.28	.770	4.17	.980	0.561
T4 (pmol/L)	15.7	2.4	15.2	2.5	0.431
TSH (mIU/L)	1.79	2.06	1.73	.92	0.097
PSA *(nmol/L)	1.95	9.06	2.33	3.38	0.115
Sleep apnea (AHI)^*+*^					**0.050**
AHI <10%	46.2%	157	37.9%	36	
AHI ≥10%	53.8%	183	62.1%	59	
Erectile Function (IIEF–5)	20.1	8.0	17.3	7.0	**0.042**
Solitary Sexual Desire *(SDI-II)	7	0.21	5	0.23	**0.006**
Dyadic Sexual Desire *(SDI-II)	48	0.52	44	0.50	**0.039**
Health conditions ^*ϕ*^					
Angina	5.3%	46	6.5%	56	0.065
Asthma	12.0%	105	11.3%	97	0.231
Diabetes	10.8%	95	16.6%	14	**0.042**
Osteoarthritis	8.6%	75	10.5%	90	0.580
Rheumatoid arthritis	4.6%	40	4.5%	39	0.777
Prostate Cancer	3.7%	26	4.8%	40	0.155

Data presented are mean & standard deviation (continuous) or percentage & number (categorical). *Non-normally distributed data are presented as median & SEM. Δ DEXA= dual-energy X-ray absorptiometry; PSA= Prostate-Specific Antigen; + AHI= Apnea-Hypopnea Index; f Health conditions were determined by participant response to question regarding multiple chronic diseases (“Have you ever been told by a doctor that you have any of the following conditions?”)

### LUTS, depression and anxiety incidence, and inflammatory markers

Table 2 detail the baseline descriptive covariates and regression estimates for incident storage and voiding LUTS. At follow-up, 16.3% (n = 108) of men reported incident storage symptoms (19 cases per 1000 person-years), compared with 12.0% (n = 88; 11 cases per 1000 person-years) for incident voiding LUTS. Men with incident storage and voiding symptoms were more likely at baseline to have both depression (17.9% (n = 26) & 27.6% (n = 24)) and anxiety (20.5% (n = 31) & 21.6% (n = 19)), respectively (Table 2).

**Table 2:** Baseline characteristics and multi-stage regression estimates for incident storage and voiding LUTS (AUA-SI) in a community-based cohort of Australian men.

	**Storage LUTS (Follow-up)**					**Multistage regression of incident Storage LUTS**														
	**No incident (n = 553)**		**Incident (n = 108)**			**Unadjusted**		**Unadjusted (35–64yrs)**		**Unadjusted (65–80yrs)**		**Multi-adjusted 1** (Full Model)*			**Multi-adjusted 2** (- inflammation)^#^			**Multi-adjusted 3** (- Anx./Depres/LUTS)^Δ^		
	**%**	**N**	**%**	**N**	***p***	**OR**	**99%CI**	**OR**	**99%CI**	**OR**	**99%CI**	**OR**	**99%CI**	***R*^*2*^**	**OR**	**99%CI**	***R*^*2*^**	**OR**	**99%CI**	***R*^*2*^**
Depression (Diagnosed/Symptomatic/Meds)	**13.5%**	**70**	**17.9%**	**26**	**0.001**	**1.49**	**(1.02, 3.17)**	0.66	(0.25, 1.74)	**1.66**	**(1.01, 2.92)**	**1.26**	**(1.01, 4.02)**	0.253	1.18	(0.96, 3.74)	0.221			
Anxiety (Diagnosed/Symptomatic/Meds)	**13.5%**	**69**	**20.5%**	**31**	**0.001**	**1.65**	**(1.03, 2.64)**	1.12	(0.45, 2.74)	**1.99**	**(1.15, 3.47)**	**1.74**	**(1.05, 2.21)**	0.253	**1.67**	**(1.02, 2.71)**	0.221			
Voiding LUTS (AUA-SI; categ.)	**4.9%**	**25**	**13.9%**	**21**	**0.001**	**3.13**	**(1.40, 5.00)**	2.02	(0.86, 4.72)	**4.23**	**(1.71, 10.49)**	**2.63**	**(1.11, 7.35)**	0.253	**1.98**	**(1.03, 4.51)**	0.221			
	***X***	**SD**	***X***	**SD**																
CRP (mg/L)	2.08	2.48	2.50	1.92	**0.048**	**1.08**	**(1.02, 1.12)**	**1.29**	**(1.09, 1.86)**	0.93	(0.65, 1.35)	1.06	(0.91, 1.96)	0.253				1.04	(0.81, 3.12)	0.187
IL–6 (pg/mL)	2.30	1.80	2.37	2.21	0.615	0.89	(0.72, 1.10)	0.99	(0.74, 1.35)	0.89	(0.57, 1.42)									
MPO (ug/L)	118.2	64.00	127.4	78.50	0.312	0.93	(0.77, 1.14)	0.89	(0.64, 1.26)	0.78	(0.44, 1.40)									
TNF-α (pg/mL)	2.257	1.516	2.335	3.462	0.711	0.92	(0.69, 1.23)	0.98	(0.73, 1.32)	0.92	(0.56, 1.51)									
e-Selectin (ng/mL)	36.08	11.98	35.80	14.77	0.812	0.83	(0.68, 1.01)	1.03	(0.76, 1.39)	0.83	(0.50, 1.37)									
	**Voiding LUTS (Follow-up)**					**Multistage regression of incident Voiding LUTS**														
	**No incident (n = 642)**		**Incident (n = 88)**			**Unadjusted**		**Unadjusted (35–64yrs)**		**Unadjusted (65–80yrs)**		**Multi-adjusted 1** (Full model)*			**Multi-adjusted 2** (- inflammation)^#^					
	**%**	**N**	**%**	**N**	***p***	**OR**	**99%CI**	**OR**	**99%CI**	**OR**	**99%CI**	**OR**	**99%CI**	***R*** ^***2***^	**OR**	**99%CI**	***R*** ^***2***^			
Depression (Diagnosed/Symptomatic/Meds)	**19.6%**	**122**	**27.6%**	**24**	**0.001**	**1.56**	**(1.02, 2.60)**	1.64	(0.64, 4.24)	**1.79**	**(1.01, 3.32)**	1.47	(0.80, 2.68)	0.193	1.43	(0.78, 2.82)	0.189			
Anxiety (Diagnosed/Symptomatic/Meds)	**13.4%**	**86**	**21.6%**	**19**	**0.040**	**1.78**	**(1.02, 3.11)**	0.65	(0.18, 2.30)	**2.78**	**(1.47, 5.31)**	1.51	(0.77, 2.95)	0.193	1.46	(0.72, 3.08)	0.189			
Storage LUTS (AUA-SI; categ.)	15.4%	99	18.2%	16	0.505	1.22	(0.57, 2.62)	1.17	(0.55, 2.50)	1.26	(0.50, 3.19)									
	***X***	**SD**	***X***	**SD**																
CRP (mg/L)	1.95	1.87	2.13	2.32	0.411	1.03	(0.81, 1.84)	1.23	(0.74, 2.03)	0.50	(0.15, 1.61)									
IL–6 (pg/mL)	2.17	1.53	2.06	2.13	0.721	0.98	(0.75, 1.21)	1.01	(0.68, 1.49)	0.94	(0.57, 1.56)									
MPO (ug/L)	124.7	77.1	143.9	106.2	0.269	1.21	(0.83, 1.58)	1.18	(0.85, 1.63)	1.37	(0.84, 2.26)									
TNF-α (pg/mL)	2.316	3.248	2.106	1.280	0.582	0.92	(0.78, 1.36)	0.80	(0.30, 2.10)	0.92	(0.50, 1.69)									
e-Selectin (ng/mL)	35.57	14.54	33.79	12.58	0.236	1.01	(0.98, 1.05)	0.99	(0.67, 1.48)	0.76	(0.44, 1.31)									

Data presented are mean & standard deviation (continuous) or percentage & number (categorical). Non-normally distributed data are presented as median & SEM.

* Model 1 includes all covariates with a univariate association with the outcome measure of p<0.25

^#^ Identical to Model 1, excluding inflammation markers with a univariate association of p<0.25.

^Δ^ Identical to Model 1, excluding LUTS, anxiety & depression. Full model also adjusted for: (Storage LUTS) age, BMI, handgrip strength, marital status, work status, household income, recreational exercise level, LDL cholesterol, total testosterone, sleep apnea (AHI>10), diabetes; (Voiding LUTS) As previous, minus work status, LDL cholesterol, also: HDL cholesterol, serum prostate specific antigen (PSA), erectile dysfunction, angina (see: S1 Table for confounder univariate and multivariate data).

CRP was higher in men with incident storage LUTS, with no other significant differences between either storage or voiding LUTS for other inflammatory markers (Table 2).

Of the other covariates, men with incident storage LUTS were found to be older, have higher BMI, plasma LDL cholesterol, SHBG, E_2_, T_4_, and PSA, with lower handgrip strength, plasma testosterone and erectile function. Men with incident storage LUTS were also more likely to be widowed, unemployed, retired, have lower physical activity, have measured sleep apnea, and a previous diagnosis of angina, anxiety, diabetes, and osteoarthritis. Men with incident voiding LUTS, were older, had higher systolic blood pressure, plasma PSA and lower handgrip strength, plasma testosterone, dyadic sexual desire and erectile function. Men with incident voiding LUTS were more likely to be widowed, unemployed, retired, have lower income, and a previous diagnosis of diabetes and osteoarthritis (S1 Table)

In unadjusted regression models of incident storage LUTS, there was a strong positive association between depression, anxiety, and voiding LUTS (in order of size of effect) at baseline. When stratified according to middle aged to older men (35–64 years) and elderly men (65–80 years), these effects were strongest in the latter group. In multi-adjusted models, depression, anxiety, and voiding LUTS at baseline maintained the positive association with the development of storage LUTS at follow-up (OR: 1.26 (1.01, 4.02), 1.74 (1.05, 2.21), and 2.63 (1.11, 7.35), respectively).

In unadjusted regression models of incident voiding LUTS, there was a comparable positive association between depression and anxiety at baseline. Again, these effects were strongest in elderly (cf. middle aged to older) men. These effects however were not maintained in multi-adjusted models of voiding LUTS.

An age-adjusted effect was observed for CRP on incident storage LUTS. No other significant effects of other inflammatory markers were observed in final models of incident storage and voiding LUTS (Table 2).

### Incident depression, anxiety, LUTS and inflammatory markers

Table 3 details the baseline descriptive covariates and regression estimates for incident depression and anxiety. At follow-up, 14.5% (n = 108) and 12.2% (n = 10) of men reported incident depression and anxiety, respectively. In unadjusted models of incident depression, there was a strong positive association between anxiety, and storage LUTS at baseline. When stratified according to middle aged to older men (35–64 years) and elderly men (65–80 years), these effects were stronger in the elderly group for anxiety and voiding LUTS, and in the younger group for storage LUTS. The effect of anxiety and storage LUTS was maintained in multi-adjusted models of incident depression (OR: 2.7; 99%CI:1.65, 7.89, and 1.45 (1.05, 2.36), respectively). For incident anxiety, there was a strong positive association between storage, voiding LUTS, and depression (in order of size of effect) at baseline. These effects were similar when the sample was stratified into middle aged to older men and elderly men. In multi-adjusted models, only the effect of depression and voiding LUTS were maintained (5.06 (2.81, 9.11) and 2.40 (1.16, 4.98), respectively).

**Table 3:** Baseline characteristics and multi-stage regression estimates for incident depression & anxiety (diagnosed/symptomatic/medications) in a community-based cohort of Australian men.

	**Depression (Follow-up)**					**Multistage regression of incident depression**														
	**No incident (n = 553)**		**Incident (n = 108)**			**Unadjusted**		**Unadjusted (35–64yrs)**		**Unadjusted (65–80yrs)**		**Multi-adjusted 1** (Full Model)*			**Multi-adjusted 2** (- inflammation)^#^			**Multi-adjusted 3** (- Anxiety/LUTS)^Δ^		
	**%**	**N**	**%**	**N**	***p***	**OR**	**99%CI**	**OR**	**99%CI**	**OR**	**99%CI**	**OR**	**99%CI**	***R*^*2*^**	**OR**	**99%CI**	***R*^*2*^**	**OR**	**99%CI**	***R*^*2*^**
Anxiety (Diagnosed/Symptomatic/Meds)	**6.9%**	**42**	**27.2%**	**25**	**0.001**	**2.56**	**(1.05, 6.28)**	2.33	(0.78, 6.89)	**4.39**	**(1.39, 13.87)**	**2.77**	**(1.65, 7.89)**	0.244	**1.82**	**(1.02, 2.71)**	0.220			
Voiding LUTS (AUA-SI; categ.)	**16.6%**	**25**	**23.9%**	**22**	**0.018**	1.59	(0.94 2.68)	1.09	(0.49, 2.42)	**2.52**	**(1.15, 5.53)**	1.18	(0.58, 2.41)	0.244						
Storage LUTS (AUA-SI; categ.)	**23.0%**	**140**	**32.6%**	**30**	**0.004**	**1.63**	**(1.10, 2.61)**	**2.84**	**(1.30, 6.19)**	1.16	(0.61, 2.21)	**1.45**	**(1.05, 2.36)**	0.244	**1.98**	**(1.03, 4.51)**	0.220			
	***X***	**SD**	***X***	**SD**																
CRP (mg/L)	2.08	2.48	2.50	1.92	**0.048**	1.13	(0.86, 1.50)	1.32	(0.92, 2.41)	0.86	(0.32, 2.31)									
IL–6 (pg/mL)	2.30	1.80	2.37	2.21	0.615	1.03	(0.72, 1.46)	1.12	(0.76, 1.66)	0.75	(0.24, 2.35)									
MPO (ug/L)	118.2	64.00	127.4	78.50	0.312	1.16	(0.83, 1.63)	1.18	(0.83, 1.68)	1.05	(0.46, 2.36)									
TNF-α (pg/mL)	2.257	1.516	2.535	3.462	**0.011**	**1.22**	**(1.10, 1.62)**	**1.27**	**(1.05, 1.90)**	**1.15**	**(1.01, 1.73)**	**1.17**	**(1.01, 1.54)**	0.244				1.13	(0.89, 1.79)	0.171
e-Selectin (ng/mL)	36.08	11.98	35.80	14.77	0.812	1.18	(0.82, 1.73)	1.14	(0.73, 1.77)	1.26	(0.57, 2.77)									
	**Anxiety (Follow-up)**					**Multistage regression of incident anxiety**														
	**No incident (n = 769)**		**Incident (n = 107)**			**Unadjusted**		**Unadjusted (35–64yrs)**		**Unadjusted (65–80yrs)**		**Multi-adjusted 1** (Full model)*			**Multi-adjusted 2** (- inflammation)^#^			**Multi-adjusted 3** (- Depression/LUTS) ^Δ^		
	**%**	**N**	**%**	**N**	***p***	**OR**	**99%CI**	**OR**	**99%CI**	**OR**	**99%CI**	**OR**	**99%CI**	***R*** ^***2***^	**OR**	**99%CI**	***R*** ^***2***^	**OR**	**99%CI**	***R*** ^***2***^
Depression (Diagnosed/Symptomatic/Meds)	**15.2%**	**113**	**44.6%**	**45**	**0.001**	**4.49**	**(2.89, 6.97)**	**4.59**	**(2.78, 7.58)**	**3.78**	**(1.46, 9.78)**	**5.06**	**(2.81, 9.11)**	0.190	**5.48**	**(2.98, 10.07)**	0.170			
Storage LUTS (AUA-SI; categ.)	**27.1%**	**208**	**35.5%**	**38**	**0.039**	**1.48**	**(1.01, 2.27)**	1.53	(0.91, 2.57)	2.01	(0.87, 4.66)	0.72	(0.36, 1.43)	0.190	0.82	(0.40, 1.69)	0.170			
Voiding LUTS (AUA-SI; categ.)	**18.2%**	**140**	**29.9%**	**32**	**0.004**	**1.91**	**(1.22, 3.01)**	**2.39**	**(1.33, 4.27)**	**2.50**	**(1.08, 5.82)**	**2.40**	**(1.16, 4.98)**	0.190	2.18	(0.96, 4.74)	0.170			
	***X***	**SD**	***X***	**SD**																
CRP (mg/L)	2.50	4.13	2.79	3.51	0.411	1.03	(0.81, 1.84)	1.21	(0.90, 1.63)	0.65	(0.22, 1.90)									
IL–6 (pg/mL)	2.33	2.11	2.30	1.50	0.721	0.98	(0.75, 1.21)	1.04	(0.76, 1.42)	0.90	(0.47, 1.74)									
MPO (ug/L)	125.0	80.2	126.6	77.0	0.269	1.21	(0.83, 1.58)	0.98	(0.70, 1.34)	1.12	(0.63, 1.99)									
TNF-α (pg/mL)	2.313	3.059	1.932	0.949	0.261	0.92	(0.78, 1.36)	0.61	(0.23, 1.61)	0.62	(0.13, 2.96)									
e-Selectin (ng/mL)	**34.77**	**13.80**	**38.80**	**16.91**	**0.009**	**1.30**	**(1.01, 1.67)**	1.22	(0.91, 1.63)	1.49	(0.88, 2.53)	**1.35**	**(1.03, 1.76)**	0.190				**1.30**	**(1.02, 1.65)**	0.081

*Data presented are mean & standard deviation (continuous) or percentage & number (categorical)*. *Non-normally distributed data are presented as median & SEM*.

* *Model 1 includes all covariates with a univariate association with the outcome measure of p<0*.*25*

^*#*^ *Identical to Model 1*, *excluding inflammation markers with a univariate association of p<0*.*25*.

^*Δ*^ *Identical to Model 1*, *excluding LUTS and depression or anxiety*. *Full model also adjusted for*: *(Depression) age category*, *BMI*, *handgrip strength*, *marital status*, *work status*, *household income*, *recreational exercise level*, *total testosterone*, *erectile function*, *solitary and dyadic sexual desire*, *sleep apnea (AHI>10)*, *angina*, *diabetes; (Anxiety) As previous*, *minus handgrip strength*, *sexual desire*, *diabetes*, *also*: *systolic BP*, *serum prostate specific antigen (PSA)*, *see*: *S* 2 *Table for confounder univariate and multivariate data)*.

TNF-α and e-Selectin was higher in men with incident depression and anxiety, respectively, at follow-up (2.535±3.462 pg/mL vs. 2.257±1.516 pg/mL at baseline; and 38.80±16.91 ng/mL vs. 34.77±13.80). There was no major difference between the unadjusted regression estimates for either TNF-α or e-Selectin on incident depression and anxiety, respectively, between middle aged to older and elderly men. These positive associations were maintained in multi-adjusted models of depression and anxiety for both TNF-α and e-Selectin (Table 3).

Of the other covariates, men with incident depression were found to be older, have higher abdominal fat mass, plasma LDL cholesterol, and E_2_, with lower handgrip strength, plasma testosterone, erectile function and solitary and dyadic sexual desire. Men with incident depression were also more likely to be widowed, unemployed, retired, lower effective retrievals in memory testing, and a previous diagnosis of angina. Men with incident anxiety were found to be older, have higher plasma PSA and gross household income, with lower erectile function, and more likely to be widowed, retired, have lower physical activity, and a previous diagnosis of angina (S2 Table).

### Effect size moderation of inflammatory markers on incident storage, voiding LUTS, depression and anxiety

To test for any moderating influence of inflammatory markers, those inflammation covariates with an unadjusted association with the dependent of p<0.25 were excluded from the final models (Model 2) and included without the exposure variables (Model 3). For incident storage LUTS, removing CRP from the final model produced a moderate decrease in the multi-adjusted effect size of depression (Model 1: 1.26 (1.01, 4.02) to Model 2: 1.18 (0.96, 3.4) and model fit (R^2^change: 0.253–0.221) (Table 2). For incident depression, removing TNF-α from multi-adjusted models decreased the size of the effect for anxiety (Model 1: 2.77 (1.65, 7.89) to Model 2: 1.82 (1.02, 2.71) and increased the observed estimate for storage LUTS (Model 1: 1.45 (1.05, 2.36) to Model 2: 1.98 (1.03, 4.51) and reduced the model fit (R^2^change: 0.244–0.220). For incident anxiety, removing e-Selectin attenuated the independent effect of voiding LUTS on anxiety (Model 1: 2.40 (1.16, 4.98) to Model 2: 2.18 (0.96, 4.74), R^2^ change: 0.190–0.170) (Table 3).

### Effect of pharmacotherapy on incident depression, anxiety, storage and voiding LUTS

Table 4 shows the effect of medication usage on the observed regression coefficients for incident storage, voiding LUTS, depression and anxiety. Men who had commenced or maintained both anti-cholinergic and diuretic use at follow-up were more likely to report incident storage LUTS. Men who had both ceased 5α-reductase inhibitor (5-ARI) prior to follow-up and deceased 5-ARI usage after baseline assessment, were found at an increased and decreased risk, respectively, of developing voiding LUTS. Men who had commenced stain usage since baseline assessment were at a decreased risk for incident storage LUTS and an increased risk for incident depression. For those men with incident cases of depression (based on self-report and symptomatic scores only), the commencement or continued use of anxiolytics, and the commencement of antidepressants, contributed significantly to the multivariable model of incident depression. Likewise, for men with incident cases of anxiety, the commencement or continued use of anxiolytics was associated with anxiety at follow-up. New or continued use of anxiolytic was also associated with the development of storage LUTS.

**Table 4:** Effect of medications at baseline (2002–5) and follow-up (2007–10) on incident storage, voiding LUTS, and common mental health disorder (CMHD; anxiety / depression) in a community-dwelling cohort of men.

Medications		Incident Storage LUTS					Incident Voiding LUTS					Incident Depression					Incident Anxiety				
		% (n)	OR	95%CI Lower	95%CI Upper	*R* ^*2*^ change^#^	% (n)	OR	95%CI Lower	95% CI Upper	*R* ^*2*^ change^#^	% (n)	OR	95%CI Lower	95%CI Upper	*R* ^*2*^ change^#^	% (n)	OR	95%CI Lower	95%CI Upper	*R* ^*2*^ change^#^
Anti-cholinergics	**Baseline**	**3.5 (23)**	**1.89**	**1.03**	**4.23**		2.0 (15)	1.46	0.81	5.21		1.8 (14)	1.15	0.85	2.22		2.5 (22)	1.63	0.84	2.31	
	+ / -	1.2 (8)	1.71	0.92	4.18	-0.07	1.2 (8)	1.23	0.75	4.26	+0.02	0.9 (7)	0.79	0.56	2.18	-0.02	1.2 (8)	1.62	0.91	2.42	+0.03
	+ / +	**2.3 (15)**	**2.09**	**1.07**	**5.23**	+0.07	0.8 (7)	1.35	0.86	4.23	+0.02	0.9 (7)	1.23	0.68	1.89	+0.02	2.3 (15)	1.89	0.81	3.23	+0.03
	- / +	**2.0 (13)**	**1.32**	**1.05**	**4.21**	+0.11	**1.5 (11)**	**1.38**	**1.02**	**3.76**	+0.08	1.1 (10)	1.35	0.72	3.12	+0.01	2.0 (13)	1.32	1.05	4.21	+0.04
	- / -	94.5 (638)	Ref				98.0 (715)	Ref				96.7 (721)	Ref				96.5 (845)	Ref			
Diuretics	**Baseline**	**6.7 (44)**	**2.11**	**1.11**	**3.24**		**5.2 (37)**	**1.56**	**1.03**	**2.11**		4.5 (33)	1.45	0.82	3.11		**4.7 (41)**	**1.39**	**1.03**	**1.89**	
	+ / -	4.5 (29)	1.98	0.81	3.76	-0.09	2.5 (18)	1.98	0.81	3.76	-0.09	1.8 (13)	1.65	0.78	3.01	-0.03	1.2 (10)	1.31	0.91	1.96	-0.05
	+ / +	**2.2 (15)**	**2.23**	**1.20**	**4.18**	+0.07	2.7 (19)	1.12	0.71	3.21	+0.07	2.7 (20)	1.24	0.91	3.09	+0.03	3.5 (31)	1.47	0.98	1.92	+0.05
	- / +	**4.3 (25)**	**1.89**	**1.10**	**2.89**	+0.18	4.3 (25)	1.69	0.91	2.27	+0.10	3.3 (23)	1.69	0.90	3.33	+0.05	**3.2 (29)**	**1.21**	**1.01**	**1.49**	**+0.06**
	- / -	90.8 (594)	Ref				94.1 (682)	Ref				93.2 (679)	Ref				93.5 (811)	Ref			
5α-reductase inhibitors	**Baseline**	2.5 (17)	1.76	0.56	4.18		**2.9 (29)**	**1.61**	**1.05**	**2.31**		1.5 (11)	1.41	0.82	4.21		2.4 (21)	1.52	0.81	3.21	
	+ / -	2.0 (13)	1.65	0.67	3.45	-0.07	**2.0 (19)**	**1.58**	**1.09**	**3.11**	-0.15	1.2 (9)	1.53	0.79	3.32	-0.02	1.0 (9)	1.48	0.89	3.11	
	+ / +	0.5 (4)	1.56	0.87	4.22	+0.07	0.9 (10)	1.71	0.92	4.18	-0.07	0.2 (2)	1.28	0.82	3.18	-0.01	1.4 (12)	1.66	0.91	3.33	
	- / +	**4.5 (28)**	**1.61**	**1.05**	**2.31**	+0.09	**4.9 (36)**	**0.89**	**0.77**	**-0.98**	+0.05	0.8 (7)	1.39	0.70	2.61	+0.03	**1.4 (12)**	**1.36**	**1.02**	**1.81**	
	- / -	90.8 (630)	Ref				95.2 (701)	Ref				97.9 (720)	Ref				96.1 (833)				
Statins	**Baseline**	**13.1 (85)**	**1.16**	**1.02**	**1.35**		12.5 (90)	1.32	0.81	1.89		11.5 (85)	1.32	0.81	1.89		11.5 (85)	1.32	0.81	1.89	
	+ / -	3.3 (21)	1.15	0.89	1.65	-0.06	3.3 (21)	1.15	0.72	1.91	-0.04	3.3 (21)	1.17	0.83	1.83	-0.05	3.3 (21)	1.17	0.83	1.83	-0.05
	+ / +	9.8 (64)	1.56	0.87	4.22	+0.08	9.8 (64)	1.49	0.87	2.23	+0.04	9.8 (64)	1.49	0.87	2.23	+0.05	9.8 (64)	1.49	0.87	2.23	+0.05
	- / +	**5.4 (38)**	**0.89**	**0.75**	**-0.98**	+0.09	5.4 (38)	1.27	0.92	3.21	+0.03	5.4 (38)	**1.21**	**1.03**	**2.21**	+0.05	5.4 (38)	1.41	0.96	2.21	+0.05
	- / -	85.0 (526)	Ref				85.2 (527)	Ref				87.1 (641)	Ref				87.1 (641)	Ref			
Anti-depressants	**Baseline**	**7.3 (47)**	1.54	0.85	3.21		6.5 (47)	1.46	0.79	4.23		**5.5 (43)**	**1.52**	**1.08**	**2.89**		7.2 (62)	1.46	1.07	1.89	
	+ / -	4.5 (29)	1.63	0.79	2.65	-0.05	3.2 (23)	1.78	0.59	4.29	-0.06	3.5 (27)	1.67	1.09	3.11	-0.17	3.6 (31)	1.28	0.91	2.21	-0.10
	+ / +	2.7 (18)	1.35	0.86	3.28	+0.05	3.3 (24)	1.16	0.70	4.33	+0.06	2.0 (15)	1.71	0.92	4.18	+0.07	3.6 (31)	1.64	1.12	2.21	+0.10
	- / +	**2.9 (20)**	**1.46**	**1.10**	**1.86**	+0.11	2.8 (20)	1.51	0.89	3.21	+0.11	3.0 (23)	1.32	0.89	6.01	+0.18	3.2 (29)	1.54	1.13	2.02	+0.11
	- / -	91.2 (593)	Ref				92.1 (668)	Ref				91.5 (704)	Ref				91.1 (790)				
Anxiolytics	**Baseline**	**5.5 (36)**	**1.43**	**1.05**	**2.15**		6.2 (45)	1.37	0.91	2.45		**6.7 (49)**	**1.32**	**1.05**	**3.11**		**3.4 (40)**	**1.41**	**1.06**	**1.81**	
	+ / -	1.5 (10)	1.68	0.56	2.84	-0.06	1.8 (11)	1.44	0.78	3.15	-0.05	2.5 (18)	1.28	0.89	4.02	-0.17	1.0 (12)	1.51	0.91	2.23	-0.08
	+ / +	**4.0 (26)**	**1.27**	**1.03**	**2.21**	+0.07	4.4 (34)	1.21	0.91	2.74	+0.05	**5.2 (31)**	**1.24**	**1.04**	**3.37**	+0.07	**2.4 (28)**	**1.32**	**1.03**	**1.87**	**+0.08**
	- / +	**3.2 (21)**	**1.35**	**1.03**	**2.38**	+0.02	2.0 (15)	1.33	0.87	2.84	+0.03	**3.2 (24)**	**1.21**	**1.06**	**4.21**	+0.18	**2.5 (21)**	**1.36**	**1.05**	**2.41**	**+0.09**
	- / -	93.2 (608)	Ref				93.2 (608)	Ref				92.1 (676)	Ref				96.1 (702)	Ref			
Anti-inflammatory	**Baseline**	4.5 (28)	1.17	0.81	1.89		4.7 (34)	1.23	0.71	2.21		4.8 (35)	1.34	0.79	2.98		4.6 (40)	1.41	0.81	2.11	
	+ / -	1.0 (6)	1.23	0.79	1.65	-0.02	1.2 (11)	1.18	0.82	2.13	-0.04	1.3 (11)	1.23	0.81	3.11	-0.07	1.1 (9)	1.62	0.72	2.24	-0.03
	+ / +	3.5 (22)	1.09	0.89	1.78	+0.02	3.5 (23)	1.29	0.81	2.35	+0.05	3.5 (24)	1.45	0.75	3.05	+0.07	3.5 (31)	1.29	0.89	2.47	+0.03
	- / +	2.5 (14)	1.32	0.78	1.79	+0.01	1.5 (12)	1.07	0.67	2.45	+0.02	2.0 (15)	1.36	0.95	3.35	+0.11	2.0 (17)	1.35	0.86	2.71	+0.02
	- / -	94.1 (623)	Ref				94.2 (686)	Ref				93.2 (695)	Ref				93.8 (808)	Ref			

*Data presented are OR (95% CI) from binomial regression of incident storage and voiding LUTS (AUA-SI; referent category*: *no storage / voiding LUTS) & incident depression and anxiety (self-reported physician diagnosis & symptomatic depression (BDI-1a) and anxiety (PHQ–9)*. *Medication usage assessed through Pharmaceutical Benefits Scheme linkage*. *Data include those men who were found to take selected medications up to 6 months prior to initial visit (baseline)*, *had ceased taking selected medications between baseline and follow-up (+/-)*, *those who had commenced taking selected medications after baseline visit and up to 6 months prior to follow-up visit (-/-)*, *and those who were found to have not used the selected medications (-/-; exposure referent category)*.

There was no effect of anti-inflammatory usage on either storage, voiding LUTS, depression or anxiety (Table 4).

## Discussion

This longitudinal study of community-dwelling men 35 years or older has demonstrated a bidirectional relationship between the development of LUTS and anxiety and depression, the effect of which is in part associated with inflammatory markers and dependent on LUTS symptom type. Incident depression and anxiety was predicted by the prior presence of storage and voiding LUTS, respectively. In the case of storage LUTS, this effect on incident depression was associated with higher TNF-α, whereas the effect of voiding LUTS on incident anxiety was independent of elevated e-Selectin. Conversely, incident storage LUTS was predicted by the presence of both anxiety and depression at baseline, an effect which showed an age-adjusted association with higher CRP.

LUTS has been shown to be associated with anxiety and depression in a number of cross-sectional studies [5] [14] [15] but the majority have not distinguished between LUTS clusters. Only one previous cross-sectional study has shown including voiding-type and post-micturition symptoms improves the predictive value for depression in men [16], although this cohort appeared to contain a relatively high amount of men with incomplete emptying (22.3% of men aged 40–80 years, compared with 9.3% of men from the current study). Recently published data from a large-scale registry study of Taiwanese men showed a higher likelihood of anxiety for storage, rather than voiding symptoms, in contrast to the present study. However, this study defined LUTS from inpatient/outpatient records, included depression in the definition of the outcome, and was only able to adjust for income and urbanisation. Interestingly, the association between anxiety / depression was highest for those men with benign prostatic hyperplasia (BPH), a condition most commonly associated with voiding LUTS.

To our knowledge, there has been only one longitudinal study exploring the relationship between storage LUTS and subsequent depression [17]. In this study of 392 South Korean men aged 65 years or older, 6.2% of men were assessed as having developed depression by multiple scales (the Geriatric Depression, Centre for Epidemiologic Studies—Depression, and Hamilton Depression Scales) at follow-up (3 ± 0.3 years), and this was positively associated with storage symptoms at baseline. We have demonstrated this association with storage LUTS in a large cohort of men as young as 35 years, including those men who were currently on anti-depressants and/or a previous diagnosis of depression.

The entire cluster of voiding symptoms has yet to be specifically examined in relation to the development of either anxiety or depression. A previous study involving a large patient registry of 16,130 Taiwanese men with BPH, demonstrated that 2.6% of these men had developed depression after one year of follow-up [18]. Our findings, based on reported urinary symptoms rather than BPH alone, extend these observations by also including men with significant anxiety at follow-up.

The association between anxiety / depression and LUTS development has been previously investigated in one cross-sectional [7] and two longitudinal studies [19] [20]. In a cross-sectional study of participants from the Boston Area Community Health (BACH) Survey, men with incomplete emptying and straining (voiding LUTS) were at a much higher risk of both mild and severe depression [7]. In a longitudinal study of community-based Chinese men aged 50–70 years, those with depressive symptoms at baseline were 2.8-times more likely to develop nocturia five years later [19]. While a longitudinal analysis of men with prostate cancer demonstrated that moderate or higher levels of depression or anxiety were associated with incident voiding symptoms, although this was confounded by prostatic surgeries. The present study extends these findings to all urinary storage symptoms (including nocturia), and includes community-based men who had also been diagnosed with and treated for anxiety.

The effect of age grouping on the development of anxiety, depression, and LUTS produced mixed results. When stratifying the sample into middle-aged to older and elderly men, the effect of storage and voiding LUTS was increased and decreased for incident anxiety and depression, respectively. This is likely a function of the higher prevalence of storage and voiding LUTS in younger and older men, respectively, observed by our group [21] and others [22] [23] [24]. In the case of both incident storage and voiding symptoms, elderly men with anxiety and depression at baseline were found to have a higher likelihood of troublesome urinary symptoms at follow-up, This concurs with the bulk of evidence that suggests anxiety / depression is more likely to present with somatic (as compared with cognitive) symptoms in elderly men [25] [26]. Given power limitations and our intent to adjust for the multiple confounders of both LUTS and anxiety / depression, we were unable to test for the differential effect of age in the final models.

Observations that elevated levels of inflammatory markers are found in patients with anxiety [27] and depression [28], the co-occurrence of anxiety / depression with inflammatory diseases and the increased risk of anxiety / depression with cytokine treatment [29, 30] have suggested that increased inflammation is associated with a higher risk of mental health disorders in men. For urinary symptoms, increased systemic inflammation (as reflected most often by higher CRP levels [31]) has been associated with an increased risk of LUTS.

Of the inflammatory markers studied in the present study, CRP, IL–6 and TNF- α have been previously shown to have an association with depression / anxiety [32] [33]. Our study also demonstrated an independent, albeit comparatively small effect, of TNF-α on incident depression / anxiety. TNF- α is known to play an important role in cognitive systems that regulate the stress response [34]. Recent studies have demonstrated that elevated TNF- α supresses the activation of the serotonin receptor subtype 2A (5-HT2A), the only sub-type thought to be involved in cognition [35]. Furthermore, patients with SSRI-resistant depression show significantly higher levels of TNF- α in comparison to healthy controls [34]. To our knowledge, this study is the first demonstration of a link between TNF- α and incident depression among the general male population. We also demonstrated elevated markers of endothelial activation (e-Selectin) in subjects with incident anxiety, independent of either LUTS, depression, or other confounders. To our knowledge, only one previous study (in diabetic women) has shown an association with circulating e-Selectin and anxiety [36]. Our findings extend these observations to men and demonstrate its utility as an additional biomarker for the development of clinically-significant anxiety.

Our study demonstrated that elevated serum CRP was one mechanism whereby anxiety / depression may lead to the development of storage LUTS. Previous studies of LUTS in men have demonstrated a small, [31], or no [37], cross-sectional association between storage LUTS and CRP in men. The CRP-mediated link between depression/anxiety and the development of LUTS was not significant after adjustment for other known confounders of LUTS (e.g. hypertension, diabetes, obesity, OSA, widowhood, elevated LDL cholesterol, lower physical activity). Our modelling confirmed this with a direct, but relatively small, effect of CRP on LUTS development in comparison to these other confounders. This indicates that recent suggestions of improving LUTS outcomes by reducing inflammation is unlikely to be effective in the absence of appropriate attention to other factors [31].

The strengths of this study include the use of repeated measures from a generally representative sample of community-dwelling men from the Australian population, the quantification of multiple inflammatory markers, the examination of both symptomatic and diagnosed anxiety and depression, and the inclusion of a wide variety of bio-psychosocial covariates to control for potential confounding effects on the outcome measures. The limitations of this study include a comparatively low sample size for sensitive measures such as cytokines, the possibility that other inflammatory markers not included may have influenced the development of the selected outcome measures, the use of the IPSS for LUTS assessment meant some urological symptoms (e.g. incontinence, pain whilst voiding) were not included, the depression scale used was not optimised for elderly populations, and the reliance on self-report measures for most chronic conditions and demographic data.

## Supporting Information

S1 Table Baseline characteristics and multi-stage regression estimates for incident storage and voiding LUTS (AUA-SI) in a community-based cohort of Australian men. Data presented are mean & standard deviation (continuous) or percentage & number (categorical). *Non-normally distributed data are presented as median & SEM. Δ Percent abdominal fat mass as measured by DEXA; LTPA as measured by the National Physical Activity Survey; All health conditions refer to previous physician diagnosis; Medication usage assessed through Pharmaceutical Benefits Scheme linkage. The overall fit for the model was R2 Storage = 0.253 & Voiding = 0.193 (Nagelkerke).
(DOCX)

S2 Table Baseline descriptive characteristics and multi-stage regression estimates for incident combined depression and combined anxiety at follow-up in a community-based cohort of Australian men. Data presented are mean & standard deviation (continuous) or percentage & number (categorical). Non-normally distributed data are presented as median & SEM. Δ Percent abdominal fat mass as measured by DEXA; LTPA as measured by the National Physical Activity Survey; All health conditions refer to previous physician diagnosis; Medication usage assessed through Pharmaceutical Benefits Scheme linkage. The overall fit for the model was R2 = Depression = 0.244 & Anxiety = 0.190 (Nagelkerke).
(DOCX)
