# Supplementary material for: Lower Urinary Tract Symptoms, Depression, Anxiety and Systemic Inflammatory Factors in Men: A Population-Based Cohort Study
Source: PLoS One. 2015 Oct 7;10(10):e0137903. doi: 10.1371/journal.pone.0137903 (PMC4622039; doi:10.1371/journal.pone.0137903)
Supplement: S1 Table — Data presented are mean & standard deviation (continuous) or percentage & number (categorical). *Non-normally distributed data are presented as median & SEM. Δ Percent abdominal fat mass as measured by DEXA; LTPA as measured by the National Physical Activity Survey; All health conditions refer to previous physician diagnosis; Medication usage assessed through Pharmaceutical Benefits Scheme linkage. The overall fit for the model was R2 Storage = 0.253 & Voiding = 0.193 (Nagelkerke). (DOCX) [file pone.0137903.s001.docx]

**S1 Table.** Baseline characteristics and multi-stage regression estimates for incident storage and voiding LUTS (AUA-SI) in a community-based cohort of Australian men.

|  | **Storage LUTS (Follow-up)** | | | | | **Multistage regression of incident Storage LUTS** | | | | **Voiding LUTS (Follow-up)** | | | | | **Multistage regression of incident Voiding LUTS** | | | |
| --- | --- | --- | --- | --- | --- | --- | --- | --- | --- | --- | --- | --- | --- | --- | --- | --- | --- | --- |
|  | **No incident (n=553)** | | **Incident (n=108)** | |  | **Univariate** | | **Multi-adjusted** (Model 1)* | | **No incident (n=642)** | | **Incident (n=88)** | |  | **Univariate** | | **Multi-adjusted** | |
|  | **% / x** | **N / SD** | **% / x** | **N / SD** | ***p*** | **OR** | **99%CI** | **OR** | **99%CI** | **% / x** | **N / SD** | **% / x** | **N / SD** | ***p*** | **OR** | **99%CI** | **OR** | **99%CI** |

| Age (years) | 52 | 10 | 56 | 11 | **0.001** | **1.48** | **(1.18, 1.87)** | 1.20 | (0.72, 1.79) | 53 | 10 | 58 | 12 | **0.001** | **1.62** | **(1.12, 2.11)** | **4.68** | **(2.01, 7.12)** |
| --- | --- | --- | --- | --- | --- | --- | --- | --- | --- | --- | --- | --- | --- | --- | --- | --- | --- | --- |
| Age Group (/10yr) |  |  |  |  | **0.012** |  |  |  |  |  |  |  |  |  |  |  |  |  |
| 35-39yrs | 9.4 | 48 | 8.4 | 11 |  | Ref |  | Ref |  | 10.3% | 66 | 5.7% | 5 | **0.001** | Ref |  | Ref |  |
| 40-49yrs | 32.7 | 167 | 18.5 | 14 |  | Ref |  | Ref |  | 31.3% | 201 | 21.6% | 19 |  | Ref |  | Ref |  |
| 50-59yrs | 31.2 | 159 | 36.4 | 40 |  | 1.28 | (0.64, 2.53) | 1.07 | (0.26, 4.44) | 33.2% | 213 | 31.8% | 28 |  | 1.06 | (0.58, 1.91) | 1.87 | (0.65, 5.36) |
| 60-69yrs | 18.4 | 94 | 25.8 | 27 |  | 1.53 | (0.75, 3.14) | 0.76 | (0.13, 4.28) | 18.4% | 118 | 17.0% | 15 |  | 1.26 | (0.67, 2.40) | 1.85 | (0.53, 6.34) |
| 70-80yrs | 8.2 | 42 | 18.5 | 14 |  | 1.41 | (0.61, 3.26) | 0.83 | (0.07, 10.71) | 6.9% | 44 | 23.9% | 21 |  | 1.40 | (0.67, 2.92) | **2.05** | **(1.55, 3.63)** |
| BMI (kg/m^2^) | 27.1 | 4.0 | 29.0 | 4.7 | **0.041** | **1.11** | **(1.03, 1.32)** | 1.08 | (0.69, 3.14) | 28.5 | 4.3 | 28.0 | 4.0 | 0.098 | 1.10 | (0.73, 2.59) |  |  |
| Abdominal fat mass *^Δ^* (%;DEXA) | 33.6 | 8.1 | 33.1 | 5.1 | 0.254 | 0.94 | (0.75, 1.18) |  |  | 33.6 | 8.1 | 34.1 | 8.5 | 0.179 | 0.94 | (0.75, 1.18) |  |  |
| Hand grip strength *(Dom.; Nm) | 50.3 | 0.32 | 43.3 | 0.34 | **0.001** | **1.08** | **(1.02, 1.15)** | 1.11 | (0.79, 1.23) | 52.3 | 9.3 | 48.8 | 9.9 | **0.045** | **1.32** | **(1.05, 1.61)** | **1.21** | **(1.04, 1.57)** |
| Systolic BP (mmHg) | 134.9 | 17.1 | 138.1 | 12.8 | 0.078 | 1.15 | (1.08, 1.33) |  |  | 135.1 | 17.0 | 140.6 | 12.4 | **0.031** | **1.21** | **(1.09, 1.42)** |  |  |
| Diastolic BP (mmHg) | 85.4 | 8.8 | 85.9 | 7.7 | 0.512 | 0.98 | (0.85, 1.12) |  |  | 85.6 | 8.8 | 85.2 | 10.1 | 0.612 | 1.01 | (0.82, 1.31) |  |  |
|  |  |  |  |  |  |  |  |  |  |  |  |  |  |  |  |  |  |  |
| Marital status |  |  |  |  | **0.042** |  |  |  |  |  |  |  |  | **0.038** |  |  |  |  |
| Married / Partner | 83.5% | 461 | 88.3% | 91 |  | Ref |  | **Ref** |  | 82.6% | 530 | 80.7% | 71 |  | Ref |  | **Ref** |  |
| Separated / Divorced | 10.0% | 55 | 2.9% | 3 |  | 1.25 | (0.85, 1.90) |  |  | 9.7% | 62 | 5.7% | 5 |  | 1.23 | (0.88, 2.01) |  |  |
| Widowed | 1.1% | 6 | 3.9% | 4 |  | **1.51** | **(1.13, 2.54)** | **2.65** | (1.26, 3.86) | 1.7% | 11 | 8.0% | 7 |  | **1.62** | **(1.21, 3.61)** | **3.82** | (1.29, 6.81) |
| Never married | 5.4% | 30 | 4.9% | 5 |  | 1.11 | (0.71, 2.11) |  |  | 5.1% | 33 | 4.5% | 4 |  | 1.18 | (0.81, 1.89) |  |  |
| Work status |  |  |  |  | **0.048** |  |  |  |  |  |  |  |  | **0.031** |  |  |  |  |
| Full time | 59.4% | 463 | 50.2% | 54 |  | Ref |  |  |  | 60.1% | 386 | 50.2% | 40 |  | Ref |  |  |  |
| Part time / Casual | 9.9% | 77 | 13.1% | 12 |  | 1.96 | (0.89, 4.77) |  |  | 9.8% | 63 | 13.1% | 4 |  | 1.68 | (0.80, 3.21) |  |  |
| Unemployed | 2.3% | 18 | 3.4% | 4 |  | **2.31** | **(1.42, 3.74)** | 1.46 | (0.64, 5.01) | 2.5% | 16 | 3.4% | 2 |  | **2.22** | **(1.31, 4.01)** | 1.38 | (0.71, 4.89) |
| Retired | 20.0% | 156 | 27.2% | 33 |  | **2.40** | **(1.18, 4.89)** | 1.32 | (0.71, 6.12) | 20.1% | 129 | 38.6% | 34 |  | **3.18** | **(1.23, 5.29)** | **4.01** | **(1.82, 7.11)** |
| Educational status |  |  |  |  | 0.858 |  |  |  |  |  |  |  |  | 0.440 |  |  |  |  |
| Bachelor or higher | 19.8% | 114 | 18.3% | 20 |  | Ref |  |  |  | 15.9% | 102 | 10.2% | 9 |  | Ref |  |  |  |
| Trade/Apprenticeship | 42.6% | 245 | 39.2% | 40 |  | 1.23 | (0.71, 2.31) |  |  | 33.8% | 217 | 29.5% | 26 |  | 1.23 | (0.71, 2.31) |  |  |
| Certificate/Diploma | 31.8% | 183 | 34.2% | 36 |  | 1.11 | (0.68, 3.11) |  |  | 24.0% | 154 | 30.7% | 27 |  | 1.11 | (0.68, 3.11) |  |  |
| Other | 4.9% | 28 | 7.4% | 8 |  | 0.72 | (0.89, 2.11) |  |  | 25.2% | 162 | 28.4% | 25 |  | 0.72 | (0.89, 2.11) |  |  |
| Household Income |  |  |  |  | **0.019** |  |  |  |  |  |  |  |  | **0.028** |  |  |  |  |
| Low | 25.7% | 140 | 31.1% | 32 |  | **1.12** | **(1.03, 1.31)** | 1.03 | (0.89, 2.11) | 27.4% | 174 | 35.6% | 31 |  | **1.18** | **(1.05, 1.42)** | 1.09 | (0.89, 2.11) |
| Middle | 52.7% | 287 | 53.4% | 55 |  | **Ref** |  |  |  | 37.7% | 239 | 43.3% | 38 |  | **Ref** |  |  |  |
| High | 21.7% | 118 | 15.5% | 16 |  | **0.89** | **(0.56, 0.95)** | **0.85** | **(0.75, 0.98)** | 34.9% | 221 | 20.7% | 18 |  | **0.81** | **(0.61, 0.98)** | **0.83** | **(0.69, 0.98)** |
|  |  |  |  |  |  |  |  |  |  |  |  |  |  |  |  |  |  |  |
| Leisure time physical activity *^€^* |  |  |  |  | **0.021** |  |  |  |  |  |  |  |  | 0.112 |  |  |  |  |
| 150 mins or more | 41.0% | 320 | 33.2% | 33 |  | **Ref** |  |  |  | 43.0% | 275 | 36.7% | 32 |  | Ref |  |  |  |
| 1-149 mins | 22.3% | 174 | 39.3% | 41 |  | **1.12** | **(1.02, 1.32)** | 1.31 | (0.89, 1.67) | 32.5% | 205 | 35.6% | 31 |  | 0.92 | (0.87, 1.52) |  |  |
| None | 36.7% | 286 | 28.9% | 24 |  | **1.28** | **(1.11, 1.45)** | 0.89 | (0.71, 1.68) | 24.6% | 155 | 27.6% | 24 |  | 1.18 | (0.78, 1.55) |  |  |
| Smoking status (current) |  |  |  |  | 0.351 |  |  |  |  |  |  |  |  | 0.423 |  |  |  |  |
| Yes | 20.9% | 163 | 18.1% | 141 |  | 1.21 | (0.79, 1.56) |  |  | 21.4% | 138 | 15.9% | 14 |  | 1.11 | (0.69, 1.71) |  |  |
| No | 79.1% | 616 | 81.5% | 630 |  | Ref |  |  |  | 78.5% | 504 | 84.1% | 74 |  | Ref |  |  |  |
|  |  |  |  |  |  |  |  |  |  |  |  |  |  |  |  |  |  |  |
| Triglycerides (mmol/L) | 1.7 | 1.2 | 2.0 | 0.7 | 0.057 | 0.99 | (0.79, 1.25) |  |  | 1.7 | 1.9 | 1.8 | 0.7 | 0.116 | 0.99 | (0.79, 1.25) |  |  |
| LDL chol. (mmol/L) | 3.5 | 0.9 | 3.9 | 1.0 | **0.011** | **1.05** | **(1.00, 1.23)** | **1.05** | **(1.00, 1.21)** | 3.5 | 0.9 | 3.4 | 0.9 | **0.011** | 1.06 | (0.87, 1.23) |  |  |
| HDL chol. (mmol/L) | 1.2 | 0.3 | 1.0 | 0.2 | 0.138 | 1.03 | (0.83, 1.27) |  |  | 1.2 | 0.3 | 1.2 | 0.2 | 0.138 | 1.03 | (0.83, 1.27) |  |  |
| Total T (nmol/L) | 17.3 | 6.4 | 16.3 | 5.6 | **0.001** | **1.10** | **(1.01, 1.35)** | 1.05 | (0.99, 1.17) | 18.7 | 6.4 | 16.2 | 5.6 | **0.001** | **1.10** | **(1.01, 1.35)** | **1.08** | **(1.01, 1.21)** |
| DHT | 1.71 | 0.82 | 1.64 | 0.69 | 0.091 | 1.14 | (0.92, 1.41) |  |  | 1.91 | 0.82 | 1.84 | 0.69 | 0.091 | 1.14 | (0.92, 1.41) |  |  |
| SHBG (nmol/L) | 33.7 | 14.9 | 37.9 | 17.0 | **0.002** | 1.16 | (0.91, 1.46) |  |  | 33.9 | 14.9 | 36.8 | 17.0 | **0.002** | 1.16 | (0.91, 1.46) |  |  |
| E_2_ (pmol/L) | 94.3 | 36.1 | 103.0 | 33.0 | **0.010** | 1.16 | (0.94, 1.45) |  |  | 94.3 | 36.1 | 99.6 | 33.0 | 0.047 | 1.16 | (0.94, 1.45) |  |  |
| T3 (pmol/L) | 4.3 | 0.8 | 4.3 | 0.8 | 0.561 | 0.98 | (0.78, 1.12) |  |  | 4.3 | 0.8 | 4.3 | 0.8 | 0.561 | 0.98 | (0.78, 1.12) |  |  |
| T4 (pmol/L) | 14.7 | 2.4 | 15.2 | 2.5 | **0.002** | 1.03 | (0.78, 1.56) |  |  | 14.7 | 2.4 | 15.2 | 2.5 | **0.002** | 1.03 | (0.78, 1.56) |  |  |
| TSH (mIU/L) | 1.8 | 1.5 | 1.8 | 1.5 | 0.097 | 1.01 | (0.85, 1.19) |  |  | 1.8 | 1.5 | 1.8 | 1.5 | 0.097 | 1.01 | (0.85, 1.19) |  |  |
| PSA *(nmol/L) | 0.89 | 0.50 | 1.02 | 0.47 | **0.001** | 1.05 | (0.86, 1.18) |  |  | 1.82 | 0.50 | 2.01 | 0.47 | **0.001** | 1.06 | (01.01, 1.18) | **1.05** | **(1.01, 1.12)** |
| Sleep apnea (AHI)*^+^* |  |  |  |  | **0.022** |  |  |  |  |  |  |  |  | 0.228 |  |  |  |  |
| AHI <10% | 47.4% | 130 | 33.3% | 18 |  | Ref |  |  |  | 46.4% | 149 | 37.0% | 17 |  | Ref |  |  |  |
| AHI ≥10% | 52.6% | 144 | 66.7% | 36 |  | **1.81** | **(1.08, 3.35)** | **1.32** | **(1.08, 1.87)** | 53.6% | 172 | 63.0% | 29 |  | 1.21 | (0.89, 3.00) |  |  |
| Erectile Function (IIEF-5) | 15.9 | 6.7 | 17.3 | 7.0 | **0.042** |  |  |  |  | 15.1 | 6.7 | 18.1 | 7.0 | **0.002** | **1.13** | **(1.06, 1.31)** | **1.15** | **(1.02, 1.31)** |
| Solitary Sexual Desire *(SDI-II) | 6 | 0.21 | 5 | 0.23 | 0.466 |  |  |  |  | 6 | 0.21 | 6 | 0.23 | 0.466 |  |  |  |  |
| Dyadic Sexual Desire *(SDI-II) | 48 | 0.50 | 44 | 0.52 | **0.039** | 1.11 | (0.89, 2.11) |  |  | 47 | 0.52 | 48 | 0.50 | 0.331 |  |  |  |  |
| Other health conditions *^φ^* |  |  |  |  |  |  |  |  |  |  |  |  |  |  |  |  |  |  |
| Angina | 3.6% | 20 | 10.7% | 11 | **0.049** | **2.19** | **(1.48, 4.86)** | 1.21 | (0.87, 2.91) | 4.5% | 29 | 9.1% | 10 | **0.047** | **2.19** | **(1.48, 4.86)** | **2.03** | (**1.13, 3.94)** |
| Asthma | 11.8% | 65 | 9.7% | 10 | 0.891 | 0.87 | (0.60, 1.63) |  |  | 11.8% | 65 | 9.7% | 10 | 0.383 | 0.89 | (0.61, 1.63) |  |  |
| Diabetes | 8.9% | 49 | 10.7% | 11 | **0.001** | 1.97 | (1.03, 2.91) | **1.56** | **(1.06, 2.21)** | 12.0% | 77 | 14.8% | 13 | 0.457 | 1.61 | (0.81, 1.91) |  |  |
| Osteoarthritis | 7.1% | 39 | 13.6% | 14 | **0.012** | 2.08 | (1.09, 3.98) |  |  | 7.3% | 47 | 10.2% | 9 | 0.337 | 2.08 | (1.09, 3.98) |  |  |
| Rheumatoid arthritis | 3.8% | 21 | 4.9% | 5 |  | 1.29 | (0.57, 3.50) |  |  | 3.6% | 23 | 5.7% | 5 | 0.336 | 1.29 | (0.57, 3.50) |  |  |
| Other Cancer | 7.1% | 55 | 8.7% | 10 | **0.423** | 1.59 | (0.89, 3.21) |  |  | 7.1% | 45 | 10.5% | 10 | 0.052 | 1.59 | (0.99, 3.21) |  |  |
| Medications*^⊕^* |  |  |  |  |  |  |  |  |  |  |  |  |  |  |  |  |  |  |
| α -adrenoblockers | 7.4 | 61 | 9.1 | 11 | 0.112 | 1.32 | (0.86, 1.34) |  |  | 7.2 | 46 | 10.1 | 8 | 0.223 | 1.02 | (0.56, 1.64) |  |  |
| Anti-cholinergics | 2.3 | 27 | 4.4 | 4 | 0.024 | 2.11 | (1.05, 3.26) | **1.89** | **(1.03, 4.23)** | 4.5 | 29 | 4.4 | 4 | 0.224 | 1.11 | (0.65, 2.26) |  |  |
| Diuretics | 11.4 | 91 | 13.9 | 15 | **0.030** | **3.01** | **(1.16, 5.68)** | **2.11** | **(1.11, 3.24)** | 14.2 | 90 | 15.9 | 15 | 0.120 | 2.01 | (0.72, 3.26) |  |  |
| 5α-reductase inhibitors | 5.5 | 43 | 4.4 | 4 | 0.311 | 1.72 | (0.42, 2.56) |  |  | 5.5 | 43 | 14.8 | 13 | **0.011** | **1.72** | **(1.42, 2.06)** | **1.61** | **(1.05, 2.31)** |

*Data presented are mean & standard deviation (continuous) or percentage & number (categorical). *Non-normally distributed data are presented as median & SEM. Δ Percent abdominal fat mass as measured by DEXA;  LTPA as measured by the National Physical Activity Survey;  All health conditions refer to previous physician diagnosis;  Medication usage assessed through Pharmaceutical Benefits Scheme linkage. The overall fit for the model was R2 Storage=0.253 & Voiding=0.193 (Nagelkerke).*
