# Supplementary material for: Lower Urinary Tract Symptoms, Depression, Anxiety and Systemic Inflammatory Factors in Men: A Population-Based Cohort Study
Source: PLoS One. 2015 Oct 7;10(10):e0137903. doi: 10.1371/journal.pone.0137903 (PMC4622039; doi:10.1371/journal.pone.0137903)
Supplement: S2 Table — Data presented are mean & standard deviation (continuous) or percentage & number (categorical). Non-normally distributed data are presented as median & SEM. Δ Percent abdominal fat mass as measured by DEXA; LTPA as measured by the National Physical Activity Survey; All health conditions refer to previous physician diagnosis; Medication usage assessed through Pharmaceutical Benefits Scheme linkage. The overall fit for the model was R2 = Depression = 0.244 & Anxiety = 0.190 (Nagelkerke). (DOCX) [file pone.0137903.s002.docx]

**S2 Table.** Baseline characteristics and multi-stage regression estimates for incident combined depression and combined anxiety in a community-based cohort of Australian men.

|  | **Combined depression (Follow-up)** | | | | | **Multistage regression of incident combined depression** | | | | **Combined anxiety (Follow-up)** | | | | | **Multistage regression of incident combined anxiety** | | | |
| --- | --- | --- | --- | --- | --- | --- | --- | --- | --- | --- | --- | --- | --- | --- | --- | --- | --- | --- |
|  | **No incident (n=578)** | | **Incident (n=104)** | |  | **Univariate** | | **Multi-adjusted** | | **No incident (n=642)** | | **Incident (n=88)** | |  | **Univariate** | | **Multi-adjusted** | |
|  | **% / x** | **N / SD** | **% / x** | **N / SD** | ***p*** | **OR** | **99%CI** | **OR** | **99%CI** | **% / x** | **N / SD** | **% / x** | **N / SD** | ***p*** | **OR** | **99%CI** | **OR** | **99%CI** |

| Age (years) | 54 | 11 | 57 | 13 | **0.001** | **1.48** | **(1.18, 1.87)** |  |  | 53 | 10 | 58 | 12 | **0.001** | **1.62** | **(1.12, 2.11)** |  |  |
| --- | --- | --- | --- | --- | --- | --- | --- | --- | --- | --- | --- | --- | --- | --- | --- | --- | --- | --- |
| Age Group (/10yr) |  |  |  |  | **0.002** |  |  |  |  |  |  |  |  | 0.111 |  |  |  |  |
| 35-39yrs | 9.1% | 59 | 13.0% | 7 |  | Ref |  | Ref |  | 8.8% | 68 | 14.0% | 15 |  | Ref |  | Ref |  |
| 40-49yrs | 28.5% | 185 | 35.2% | 19 |  | 1.14 | (0.51, 2.53) | 1.11 | (0.44, 2.80) | 28.1% | 216 | 32.7% | 35 |  | 1.85 | (0.82, 4.15) | 1.35 | (0.45, 4.03) |
| 50-59yrs | 29.0% | 188 | 22.2% | 12 |  | 0.78 | (0.34, 1.80) | 0.76 | (0.29, 1.99) | 29.0% | 223 | 30.8% | 33 |  | 1.24 | (0.51, 2.99) | 1.61 | (0.57, 4.58) |
| 60-69yrs | 21.6% | 140 | 9.3% | 5 |  | 0.42 | (0.16, 1.10) | 0.37 | (0.12, 1.14) | 21.1% | 162 | 15.0% | 16 |  | 0.76 | (0.11, 2.86) | 0.85 | (0.27, 2.66) |
| 70-80yrs | 11.7% | 76 | 20.4% | 11 |  | **2.02** | **(1.12, 4.75)** | **1.18** | **(1.02, 2.40)** | 13.0% | 100 | 7.5% | 8 |  | 2.03 | (0.91, 4.53) | **3.74** | **(1.10, 12.67)** |
| BMI (kg/m^2^) | 28.4 | 4.1 | 30.4 | 4.3 | **0.041** | **1.11** | **(1.03, 1.32)** | 1.08 | (0.69, 3.14) | 28.5 | 4.3 | 28.6 | 4.5 | 0.118 | 1.04 | (0.85, 1.28) |  |  |
| Abdominal fat mass *^Δ^* (%;DEXA) | 33.6 | 7.7 | 34.9 | 7.6 | 0.254 | 0.94 | (0.75, 1.18) |  |  | 33.8 | 8.1 | 34.1 | 7.2 | 0.179 | 1.07 | (0.83, 1.29) |  |  |
| Hand grip strength *(Dom.; Nm) | 50.14 | 9.64 | 47.04 | 9.97 | **0.001** | **0.84** | **(0.71, 0.95)** | **0.87** | **(0.75, 0.98)** | **46.8** | **9.6** | **49.0** | **8.8** | **0.045** | 0.89 | (0.65, 1.11) |  |  |
| Systolic BP (mmHg) | 136.67 | 17.71 | 137.53 | 18.72 | 0.078 | 1.15 | (1.08, 1.33) |  |  | 137.0 | 17.8 | 137.8 | 21.7 | 0.631 | **1.21** | **(1.09, 1.42)** | 1.08 | (0.91, 1.54) |
| Diastolic BP (mmHg) | 85.43 | 8.82 | 85.43 | 9.73 | 0.512 | 0.98 | (0.85, 1.12) |  |  | **85.1** | **8.7** | **87.6** | **10.3** | **0.012** | 1.01 | (0.82, 1.31) |  |  |
|  |  |  |  |  |  |  |  |  |  |  |  |  |  |  |  |  |  |  |
| Marital status |  |  |  |  | 0.695 |  |  |  |  |  |  |  |  | **0.038** |  |  |  |  |
| Married / Partner | 85.1% | 418 | 85.9% | 79 |  | Ref |  | **Ref** |  | 84.1% | 645 | 82.2% | 88 |  | Ref |  | **Ref** |  |
| Separated / Divorced | 8.4% | 51 | 8.7% | 8 |  | 1.25 | (0.85, 1.90) |  |  | 9.1% | 70 | 9.3% | 10 |  | 1.23 | (0.88, 2.01) |  |  |
| Widowed | 2.1% | 13 | 3.3% | 3 |  | **1.51** | **(1.13, 2.54)** | **2.65** | (1.26, 3.86) | 2.5% | 19 | 7.4% | 9 |  | **1.62** | **(1.21, 3.61)** | **3.82** | **(1.29, 6.81)** |
| Never married | 4.4% | 27 | 2.2% | 2 |  | 1.11 | (0.71, 2.11) |  |  | 4.3% | 33 | 5.6% | 6 |  | 1.18 | (0.81, 1.89) |  |  |
| Work status |  |  |  |  | **0.017** |  |  |  |  |  |  |  |  | **0.031** |  |  |  |  |
| Full time | 65.0% | 343 | 56.4% | 70 |  | Ref |  |  |  | 55.7% | 428 | 58.9% | 63 |  | Ref |  |  |  |
| Part time / Casual | 2.1% | 14 | 2.5% | 5 |  | 1.96 | (0.89, 4.77) |  |  | 8.7% | 67 | 10.3% | 11 |  | 1.68 | (0.80, 3.21) |  |  |
| Unemployed | 6.7% | 46 | 6.7% | 12 |  | **2.31** | **(1.42, 3.74)** | 1.46 | (0.64, 5.01) | 2.1% | 16 | 9.2% | 10 |  | **2.22** | **(1.31, 4.01)** | 1.38 | (0.71, 4.89) |
| Retired | 26.2% | 179 | 27.9% | 28 |  | **2.40** | **(1.18, 4.89)** | 1.32 | (0.71, 6.12) | 27.1% | 208 | 17.8% | 19 |  | **3.18** | **(1.23, 5.29)** | **4.01** | **(1.82, 7.11)** |
| Educational status |  |  |  |  | 0.973 |  |  |  |  |  |  |  |  | 0.440 |  |  |  |  |
| Bachelor or higher | 13.1% | 80 | 12.0% | 11 |  | Ref |  |  |  | 14.0% | 108 | 13.1% | 14 |  | Ref |  |  |  |
| Trade/Apprenticeship | 34.1% | 208 | 35.9% | 33 |  | 1.23 | (0.71, 2.31) |  |  | 33.8% | 260 | 32.7% | 35 |  | 1.23 | (0.71, 2.31) |  |  |
| Certificate/Diploma | 24.4% | 149 | 26.1% | 24 |  | 1.11 | (0.68, 3.11) |  |  | 25.5% | 196 | 26.2% | 28 |  | 1.11 | (0.68, 3.11) |  |  |
| Other | 27.5% | 28 | 25.0% | 23 |  | 0.72 | (0.89, 2.11) |  |  | 25.9% | 199 | 26.2% | 28 |  | 0.72 | (0.89, 2.11) |  |  |
| Household Income |  |  |  |  | **0.044** |  |  |  |  |  |  |  |  | **0.028** |  |  |  |  |
| Low | 29.6% | 199 | 34.7% | 36 |  | **1.12** | **(1.03, 1.31)** | 1.03 | (0.89, 2.11) | 27.4% | 174 | 35.6% | 31 |  | **1.18** | **(1.05, 1.42)** | 1.09 | (0.89, 2.11) |
| Middle | 51.0% | 243 | 51.5% | 53 |  | **Ref** |  |  |  | 37.7% | 239 | 43.3% | 38 |  | **Ref** |  |  |  |
| High | 19.3% | 130 | 13.9% | 14 |  | **0.89** | **(0.56, 0.95)** | **0.85** | **(0.75, 0.98)** | 34.9% | 221 | 20.7% | 18 |  | **0.81** | **(0.61, 0.98)** | **0.83** | **(0.69, 0.98)** |
|  |  |  |  |  |  |  |  |  |  |  |  |  |  |  |  |  |  |  |
| Leisure time physical activity *^€^* |  |  |  |  | **0.071** |  |  |  |  |  |  |  |  | **0.112** |  |  |  |  |
| 150 mins or more | 42.9% | 238 | 34.3% | 36 |  | **Ref** |  |  |  | 41.4% | 313 | 29.6% | 31 |  | **Ref** |  | **Ref** |  |
| 1-149 mins | 32.6% | 179 | 36.3% | 38 |  | **1.12** | **(1.02, 1.32)** | 1.31 | (0.89, 1.67) | 33.9% | 257 | 32.4% | 34 |  | **1.52** | **(1.05, 2.52)** | **1.15** | **(0.91, 2.71)** |
| None | 24.6% | 165 | 29.4% | 30 |  | **1.28** | **(1.11, 1.45)** | 0.89 | (0.71, 1.68) | 24.7% | 187 | 38.1% | 40 |  | **2.18** | **(1.78, 2.55)** | **2.43** | **(1.26, 3.21)** |
| Smoking status (current) |  |  |  |  | **0.022** |  |  |  |  |  |  |  |  | 0.423 |  |  |  |  |
| Yes | 18.6% | 127 | 26.0% | 28 |  | 1.21 | (0.79, 1.56) |  |  | 16.3% | 125 | 24.3% | 26 |  | 1.11 | (0.69, 1.71) |  |  |
| No | 81.4% | 455 | 74.0% | 76 |  | Ref |  |  |  | 80.6% | 620 | 72.0% | 77 |  | Ref |  |  |  |
| Fuld Object Memory Evaluation |  |  |  |  |  |  |  |  |  |  |  |  |  |  |  |  |  |  |
| Ineffective reminders | 3.9 | 3.2 | 4.3 | 3.8 | 0.236 | 1.06 | (0.89, 1.31) |  |  | 3.3 | 2.7 | 3.7 | 2.5 | 0.658 | 1.01 | (0.76, 1.45) |  |  |
| Repeated retrievals | 25.2 | 11.5 | 20.6 | 12.3 | **0.011** | **0.76** | **(0.53, 0.91)** | 0.81 | (0.49, 1.11) | 24.7 | 12.3 | 21.1 | 11.8 | 0.068 | **0.89** | **(0.71, 0.91)** | 0.88 | (0.65, 1.05) |
| Trail Making Test |  |  |  |  |  |  |  |  |  |  |  |  |  |  |  |  |  |  |
| A (secs) | 15.3 | 6.7 | 15.1 | 7.2 | 0.298 | 1.08 | (0.71, 1.39) |  |  | 15.8 | 6.2 | 15.5 | 7.0 | 0.366 | 1.05 | (0.86, 1.57) |  |  |
| B (secs) | 81.3 | 21.8 | 84.7 | 26.8 | 0.214 | 1.11 | (0.68, 1.67) |  |  | 80.2 | 23.1 | 81.4 | 25.8 | 0.364 | 1.19 | (0.88, 1.43) |  |  |
| Triglycerides (mmol/L) | 1.68 | 1.19 | 1.74 | 1.32 | 0.057 | 0.99 | (0.79, 1.25) |  |  | 1.7 | 1.2 | 1.9 | 1.3 | 0.116 | 0.99 | (0.79, 1.25) |  |  |
| LDL chol. (mmol/L) | 3.5 | .9 | 3.9 | 1.1 | **0.011** | **1.05** | **(1.00, 1.23)** |  |  | 3.5 | 0.9 | 3.4 | 1.0 | 0.611 | 1.06 | (0.87, 1.23) |  |  |
| HDL chol. (mmol/L) | 1.2 | .3 | 1.2 | .3 | 0.138 | 1.03 | (0.83, 1.27) |  |  | 1.2 | 0.3 | 1.2 | 0.3 | 0.738 | 1.03 | (0.83, 1.27) |  |  |
| Total T (nmol/L) | 17.61 | 6.19 | 17.01 | 5.43 | 0.110 | **0.88** | **(0.76, 0.98)** | **0.89** | **(0.74, 0.99)** | 17.3 | 5.8 | 15.2 | 6.3 | 0.062 | 0.91 | (0.69, 1.21) |  |  |
| DHT | 1.80 | .81 | 1.68 | .70 | 0.091 | 1.14 | (0.92, 1.41) |  |  | 1.70 | 0.72 | 1.82 | 0.86 | 0.091 | 1.14 | (0.92, 1.41) |  |  |
| SHBG (nmol/L) | 33.47 | 13.64 | 33.18 | 13.97 | 0.251 | 1.16 | (0.91, 1.46) |  |  | 34.8 | 15.0 | 32.3 | 12.8 | 0.122 | 1.16 | (0.91, 1.46) |  |  |
| E_2_ (pmol/L) | 95.14 | 36.71 | 96.13 | 32.08 | 0.410 | 1.16 | (0.94, 1.45) |  |  | 95.7 | 36.8 | 88.1 | 31.8 | 0.147 | 1.16 | (0.94, 1.45) |  |  |
| T3 (pmol/L) | 4.276 | .770 | 4.168 | .980 | 0.561 | 0.98 | (0.78, 1.12) |  |  | 4.3 | 0.8 | 4.3 | 0.8 | 0.561 | 0.98 | (0.78, 1.12) |  |  |
| T4 (pmol/L) | 14.7 | 2.4 | 15.2 | 2.5 | **0.002** | 1.03 | (0.78, 1.56) |  |  | 14.7 | 2.4 | 15.2 | 2.5 | **0.002** | 1.03 | (0.78, 1.56) |  |  |
| TSH (mIU/L) | 1.79 | 2.06 | 1.73 | .92 | 0.097 | 1.01 | (0.85, 1.19) |  |  | 1.8 | 1.5 | 1.8 | 1.5 | 0.097 | 1.01 | (0.85, 1.19) |  |  |
| PSA *(nmol/L) | 2.03 | 0.67 | 2.23 | 4.40 | **0.001** | 1.05 | (0.86, 1.18) |  |  | 1.98 | 0.50 | 1.70 | 0.47 | 0.111 | 1.06 | (01.01, 1.18) | **1.05** | **(1.01, 1.12)** |
| Sleep apnea (AHI)*^+^* |  |  |  |  | **0.050** |  |  |  |  |  |  |  |  | 0.228 |  |  |  |  |
| AHI <10% | 46.2% | 157 | 37.9% | 36 |  | Ref |  | **Ref** |  | 46.4% | 149 | 37.0% | 17 |  | Ref |  |  |  |
| AHI ≥10% | 53.8% | 183 | 62.1% | 59 |  | **1.81** | **(1.08, 3.35)** | **1.32** | **(1.08, 1.87)** | 53.6% | 172 | 63.0% | 29 |  | 1.21 | (0.89, 3.00) |  |  |
| Erectile Function (IIEF-5) | 20.1 | 8.0 | 17.3 | 7.0 | **0.042** | 0.68 | (0.59, 0.80) | **0.78** | **(0.67, 0.90)** | 15.1 | 6.7 | 18.1 | 7.0 | **0.002** | **1.13** | **(1.06, 1.31)** | **1.15** | **(1.02, 1.31)** |
| Solitary Sexual Desire *(SDI-II) | 7 | 0.21 | 5 | 0.23 | **0.006** | **0.89** | **(0.65, 0.98)** | **0.91** | **(0.71, 0.99)** | 6 | 0.21 | 6 | 0.23 | 0.466 |  |  |  |  |
| Dyadic Sexual Desire *(SDI-II) | 48 | 0.52 | 44 | 0.50 | **0.039** | **0.80** | **(0.68, 0.94)** | 0.86 | (0.64, 1.08) | 47 | 0.52 | 48 | 0.50 | 0.331 |  |  |  |  |
| Other health conditions *^φ^* |  |  |  |  |  |  |  |  |  |  |  |  |  |  |  |  |  |  |
| Angina | 4.3% | 26 | 8.7% | 8 | 0.065 | **2.19** | **(1.48, 4.86)** | **2.03** | (**1.13, 3.94)** | 4.8% | 37 | 8.4% | 9 | **0.047** | **2.19** | **(1.48, 4.86)** | **2.03** | (**1.13, 3.94)** |
| Asthma | 10.3% | 63 | 16.3% | 15 | 0.089 | 0.87 | (0.60, 1.63) |  |  | 11.4% | 88 | 15.9% | 17 | 0.383 | 0.89 | (0.61, 1.63) |  |  |
| Diabetes | 9.3% | 57 | 15.2% | 14 | **0.042** | 1.97 | (0.81, 1.91) | 1.68 | (0.78, 2.21) | 10.7% | 82 | 12.1% | 13 | 0.457 | 1.61 | (0.81, 1.91) |  |  |
| Osteoarthritis | 8.2% | 50 | 6.5% | 6 | 0.580 | 2.08 | (1.09, 3.98) |  |  | 8.2% | 63 | 11.2% | 12 | 0.337 | 2.08 | (1.09, 3.98) |  |  |
| Rheumatoid arthritis | 4.8% | 29 | 5.4% | 5 | 0.777 | 1.29 | (0.57, 3.50) |  |  | 4.6% | 35 | 4.7% | 5 | 0.336 | 1.29 | (0.57, 3.50) |  |  |
| Other Cancer | 4.8% | 29 | 6.6% | 6 | 0.455 | 1.59 | (0.89, 3.21) |  |  | 7.1% | 45 | 10.5% | 10 | 0.052 | 1.59 | (0.99, 3.21) |  |  |

*Data presented are mean & standard deviation (continuous) or percentage & number (categorical). Non-normally distributed data are presented as median & SEM. Δ Percent abdominal fat mass as measured by DEXA;  LTPA as measured by the National Physical Activity Survey;  All health conditions refer to previous physician diagnosis;  Medication usage assessed through Pharmaceutical Benefits Scheme linkage. The overall fit for the model was R2=Depression=0.244 & Anxiety=0.190 (Nagelkerke).*
